# Homœopathy in the University of Michigan

**Published:** 1867-04

**Authors:** 


### Homœopathy in the University of Michigan.

It appears that the recent action of the Michigan Legislature is such, that the University at Ann Harbor cannot receive their usual appropriation from the State until a Chair of Homœopathy has been appointed. A Chair of Homœopathy in a college teaching rational medicine (?) We should like to know what principles and what practice could be agreed upon as homœopathic. We have recently been examining journals devoted to this subject; and of all the imbecile and senseless statements ever placed in type, they furnish those which stand at the head. Homœopathy taught in the same school with rational medicine (?) or, indeed, anywhere. If a man is incapable of being or doing anything else, he can take up Homœopathy without any instruction. It seems to us, that when he has been in any measure instructed, he cannot longer believe in the vagaries of such a delusion. We never had any great respect for a State Medical College, for we knew it must be subjected to influences, fatal to the cultivation of science, still this institution has prospered much beyond any reasonable expectation. Its medical staff has shown itself worthy and capable, has been of men distinguished as teachers and practitioners. This last act of folly on the part of the State Legislature, is sufficient; not anything more is required; the whole history of this institution is now in all probability written.

